# Insights into the catalysis of a lysine-tryptophan bond in bacterial peptides by a SPASM domain radical *S*-adenosylmethionine (SAM) peptide cyclase

**DOI:** 10.1074/jbc.M117.783464

**Published:** 2017-05-05

**Authors:** Alhosna Benjdia, Laure Decamps, Alain Guillot, Xavier Kubiak, Pauline Ruffié, Corine Sandström, Olivier Berteau

**Affiliations:** From the ‡ Micalis Institute, ChemSyBio, INRA, AgroParisTech, Université Paris-Saclay, 78350 Jouy-en-Josas, France and; the § Department of Molecular Sciences, Uppsala BioCenter, Swedish University of Agricultural Sciences, P. O. Box 7015, Uppsala 750-07, Sweden

**Keywords:** biosynthesis, enzyme catalysis, enzyme mechanism, iron-sulfur protein, metalloenzyme, peptide biosynthesis, radical, radical SAM, radical AdoMet, radical SAM enzyme

## Abstract

Radical *S*-adenosylmethionine (SAM) enzymes are emerging as a major superfamily of biological catalysts involved in the biosynthesis of the broad family of bioactive peptides called ribosomally synthesized and post-translationally modified peptides (RiPPs). These enzymes have been shown to catalyze unconventional reactions, such as methyl transfer to electrophilic carbon atoms, sulfur to C_α_ atom thioether bonds, or carbon-carbon bond formation. Recently, a novel radical SAM enzyme catalyzing the formation of a lysine-tryptophan bond has been identified in *Streptococcus thermophilus*, and a reaction mechanism has been proposed. By combining site-directed mutagenesis, biochemical assays, and spectroscopic analyses, we show here that this enzyme, belonging to the emerging family of SPASM domain radical SAM enzymes, likely contains three [4Fe-4S] clusters. Notably, our data support that the seven conserved cysteine residues, present within the SPASM domain, are critical for enzyme activity. In addition, we uncovered the minimum substrate requirements and demonstrate that KW cyclic peptides are more widespread than anticipated, notably in pathogenic bacteria. Finally, we show a strict specificity of the enzyme for lysine and tryptophan residues and the dependence of an eight-amino acid leader peptide for activity. Altogether, our study suggests novel mechanistic links among SPASM domain radical SAM enzymes and supports the involvement of non-cysteinyl ligands in the coordination of auxiliary clusters.

## Introduction

Radical SAM enzymes constitute one of the most diverse and versatile superfamily of enzymes, with more than 220,000 enzymes involved in at least 85 biochemical transformations (1–3). These enzymes catalyze chemically challenging reactions, some of which have no counterparts in biology or synthetic chemistry (1, 4). Recently, they have been shown to be key catalysts in the biosynthesis of the broad family of natural products called ribosomally synthesized and post-translationally modified peptides (RiPPs).^3^ RiPPs encompass major and diverse families of antibiotics and bacterial toxins, such as lantibiotics, thiopeptides, sactipeptides, and proteusins (5). In the last five years, radical SAM enzymes have been shown to catalyze unique post-translational modifications on RiPPs, such as C_α_-thioether (6, 7), carbon-carbon bond formation (8, 34), and various methyl transfer reactions on RiPPs (9, 10).

**Figure 1.**
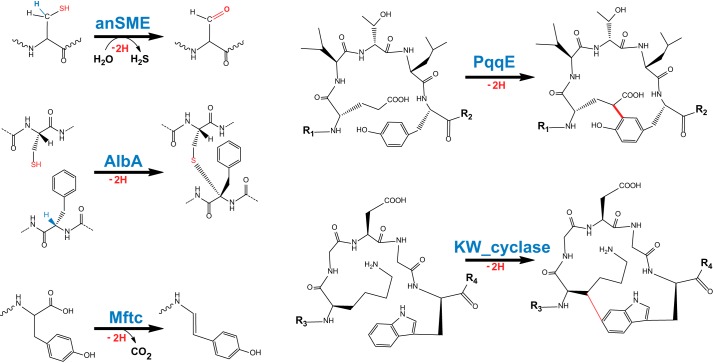
**Reactions catalyzed by SPASM domain radical SAM enzymes.** Shown are anSME (cysteine or serine oxidation into C_α_-formylglycine), AlbA (thioether bond formation), MftC (oxidative decarboxylation), PqqE (C–C bond formation), and KW_cyclase (C–C bond formation).

Interestingly, several of these post-translational modifications are catalyzed by structurally related radical SAM enzymes belonging to the so-called “SPASM domain” group. Initially this group was named for subilitosin, PQQ, anaerobic sulfatases, and mycofactocin (11, 12), the products of the reactions catalyzed by the respective enzymes AlbA (6, 7), PqqE (13), anaerobic sulfatase-maturating enzyme (anSME) (14–16), and MftC (17). AnSME is the founding member of the SPASM domain radical SAM enzyme family. It has been shown to catalyze the post-translational modification of a critical serine or cysteine residue into a C_α_-formylglycine, required for the activity of the so-called aryl sulfatases (3, 11, 14, 15, 18–21) (Fig. 1). After some controversy, it has been established that anSME, in addition of the radical SAM [4Fe-4S] cluster, contains two auxiliary [4Fe-4S] clusters in the SPASM domain (11, 20). These two [4Fe-4S] clusters are fully coordinated by eight cysteine residues, conserved in all anSME homologs (15, 19, 20, 22).

The second SPASM domain radical SAM enzyme characterized was AlbA. AlbA catalyzes the formation of C_α_-S bonds in the antibacterial peptide subtilosin A (Fig. 1) (6, 7). Intriguingly, this enzyme has seven cysteine residues in its SPASM domain that are conserved with anSME. A recent study pointed out that they are likely involved in the coordination of two auxiliary [4Fe-4S] clusters and that they are required for enzyme activity (6). More recently, PqqE has been shown to catalyze the formation of a C–C bond between a glutamate and a tyrosine residue during the biosynthesis of pyrroloquinoline quinone (13). An early study proposed that PqqE contains a single [4Fe-4S] cluster in its SPASM domain (23). However, similarly to AlbA (6), it is now suggested that PqqE possesses two auxiliary [4Fe-4S] clusters (13), likely coordinated by seven cysteine residues. Finally, recent investigations of MftC (mycofactocin biosynthesis), the “last” SPASM domain radical SAM enzyme, showed that it catalyzes tyrosine decarboxylation (17). However, its mechanism and number of [4Fe-4S] clusters are currently unclear.

All of these enzymes catalyze related reactions (Fig. 1) and have in common that they possess, in their C-terminal region, a SPASM domain that contains [4Fe-4S] clusters. However, the nature, number, and function of these metallic centers are still not well understood (21). Investigated recently, these enzymes appear now to constitute one of the largest groups in the superfamily of radical SAM enzymes. It is thus not surprising that novel SPASM domain radical SAM enzymes are regularly identified.

Recently, the KW_cyclase from *Streptococcus thermophilus* has been shown to catalyze C–C bond formation between a lysine and a tryptophan residue during the biosynthesis of a unique cyclic peptide with unknown function (8, 24). Here we investigated the substrate specificity and the role of conserved cysteine residues in the KW_cyclase from *S. thermophilus* to gain further insights into its mechanism and the large family of SPASM domain radical SAM enzymes.

## Results

### Substrate specificity of the KW_cyclase

**Figure 2.**
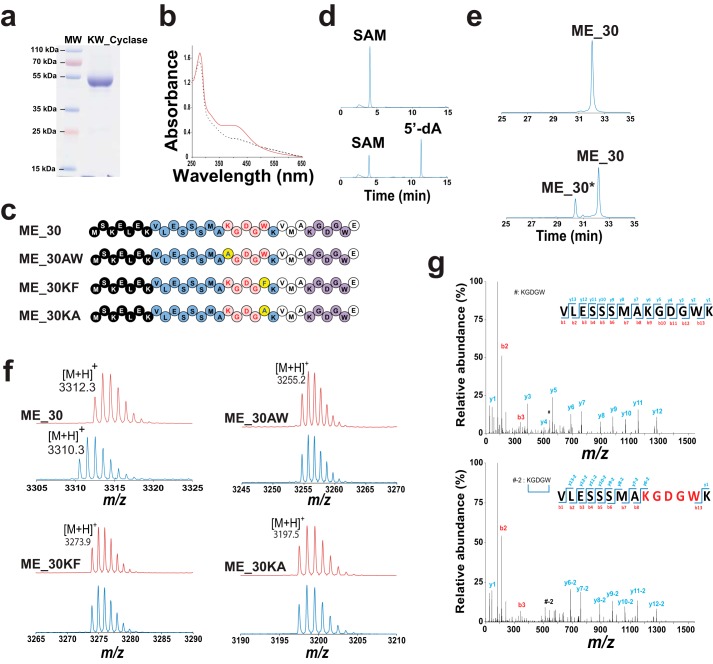
**Specificity of the KW_cyclase.** *a*, gel electrophoresis analysis (SDS-PAGE, 12.5%) of the purified KW_cyclase. *MW*, molecular weight. *b*, UV-visible spectrum of KW_cyclase before (16 μm, *dashed line*) and after (18 μm, *solid line*) anaerobic reconstitution of the [Fe-S] clusters. *c*, sequences of the ME_30, ME_30AW, ME_30KF, and ME_30KA peptides. *Black circles* correspond to the putative peptide leader based on the alignment in Fig. 3*a*. The conserved residues are depicted as *blue circles. Red circles* indicate the residues that form the cyclic KGDGW peptide. The second KGDGW motif is depicted as *purple circles*. Mutated residues are indicated by a *yellow circle. d*, HPLC analysis of the reductive cleavage of SAM at time 0 (*top trace*) and after 180-min incubation with KW_cyclase (*bottom trace*) (see below for experimental conditions). Detection was performed at 257 nm, and 5′-dA was further analyzed by MS. *e*, HPLC analysis of the ME_30 peptide before (*top trace*) and after 180-min incubation with the KW_cyclase (*bottom trace*). Detection was performed at 280 nm (see below for experimental conditions). *f*, MALDI-TOF MS analysis of the various peptides used as substrate before (*top red traces*) and after (*bottom blue traces*) incubation with KW_cyclase. Reactions were performed by incubating the reconstituted KW_cyclase (50 μm) with peptide (1 mm), SAM (1 mm), DTT (3 mm), and sodium dithionite (2 mm) under anaerobic conditions. *g*, LC/MS-MS analysis of the ME_30 peptide before (*top trace*) and after (*bottom trace*) incubation with KW_cyclase.

The nucleotide sequence coding for the KW_cyclase of *S. thermophilus* was optimized for expression in *Escherichia coli* and the protein expressed as a Strep-tag fusion protein (Fig. 2*a*). As shown (Fig. 2*b*), the purified enzyme exhibited absorption bands at ∼320 and ∼410 nm after anaerobic iron-sulfur cluster reconstitution and an *A*_420_/*A*_280_ ratio of ∼0.3, similar to what has been reported for anSME, a radical SAM enzyme containing three [4Fe-4S] clusters (15). Iron-sulfur quantification showed that the KW_cyclase contained 11.8 ± 0.3 mol of iron and 13.6 ± 0.8 mol of sulfide per polypeptide. Collectively, these data support the presence of up to three [4Fe-4S] clusters in the enzyme.

The activity of the KW_cyclase was first assayed with a peptide encompassing the 30-amino acid residues (called here ME_30; Fig. 2*c*) encoded by the *ster*_*1357* gene, as reported recently (8). Incubation of the ME_30 peptide in the presence of SAM and sodium dithionite led to the reductive cleavage of SAM into 5′-dA (retention time, 11.3 min, Fig. 2*d*) and the formation of a peptide product (ME_30*; retention time, 30.4 min; Fig. 2*e*). MS analysis of this novel peptide ([M+H]^+^, 3310.3) indicated a mass loss of −2 Da compared with the substrate ([M+H]^+^, 3312.3; Fig. 2*f*). This result, implying the loss of two H atoms, was consistent with the activity of the enzyme as a peptide cyclase. LC/MS fragmentation of tryptic peptides confirmed the formation of a bond between the expected amino acid residues: Lys-16 and Trp-20, as demonstrated by the characteristic ions *y*_6_-2 and *b*_13_ (Fig. 2*g* and supplemental Figs. S1 and S2 and Tables S1–S3).

To assess whether this enzyme can tolerate amino acid substitution, we synthesized three peptide variants in which we substituted the residues Lys-16 and Trp-20 involved in the formation of the K-W bridge (Fig. 2*c*). The KW_cyclase has been shown to abstract one lysine C_β_ H atom, generating a carbon-centered radical that is likely to perform a radical addition to the indole ring of tryptophan. We thus substituted Lys-16 with an alanine residue (peptide ME_30AW) or Trp-20 with a phenylalanine (peptide ME_30KF). Finally, as a control, we synthesized a substrate in which Trp-20 was substituted with an alanine residue (peptide ME_30KA) to prevent the radical addition of the carbon-centered radical intermediate (Fig. 2*c*). However, in all of these peptides, we conserved the second KGDGW motif present at the C-terminal end of the ME_30 peptide.

Incubation of each of these peptides with the KW_Cyclase led to efficient reductive cleavage of SAM; nevertheless, HPLC and mass spectrometry analyses failed to show any peptide modification, even after an extended incubation time (Fig. 2*f*). The absence of activity on ME_30AW and ME_30KF showed that the enzyme exhibits a strict specificity for the two amino acid residues involved in the formation of the C–C bond. This result is in contrast with the SPASM domain radical SAM enzymes anSME, AlbA, and SkfB, which tolerated amino acid substitutions in their respective substrates (7, 19, 25). Interestingly, substitution of tryptophan with a phenylalanine residue also hindered the reaction despite the fact that, following H atom abstraction, radical addition has been postulated to occur on the benzene ring of tryptophan. Hence, the enzyme appears strictly specific to the Lys and Trp residues and of the relative location of the KGDGW motif in the sequence. Indeed, despite the presence of a second KGDGW motif in the peptides assayed (*purple sequence*, Fig. 2*c*), this second motif was never modified.

### Identification of the minimum KW_cyclase peptide substrate

To further explore the enzyme specificity, we searched for genes encoding homologs of the ME_30 peptide in sequenced genomes. Genes encoding peptides are notoriously difficult to predict. Hence, we used the KW_cyclase as a probe and searched for putative peptide-coding genes in the vicinity of the gene coding for the KW_cyclase. Using a cutoff E value of E^−58^, we identified putative KW_cyclases in the genomes of several other *Streptococcus* species (*Streptococcus salivarius*, *Streptococcus agalactiae*, *Streptococcus suis*, and *Streptococcus mitis*) and in *Lactococcus lactis* as reported previously but also in *Pseudomonas putida*. Interestingly, below this E value, the next homologs retrieved were annotated as PqqE, with the authentic PqqE enzyme from *Klebsiella* among them (E value, E^−14^) (23).

**Figure 3.**
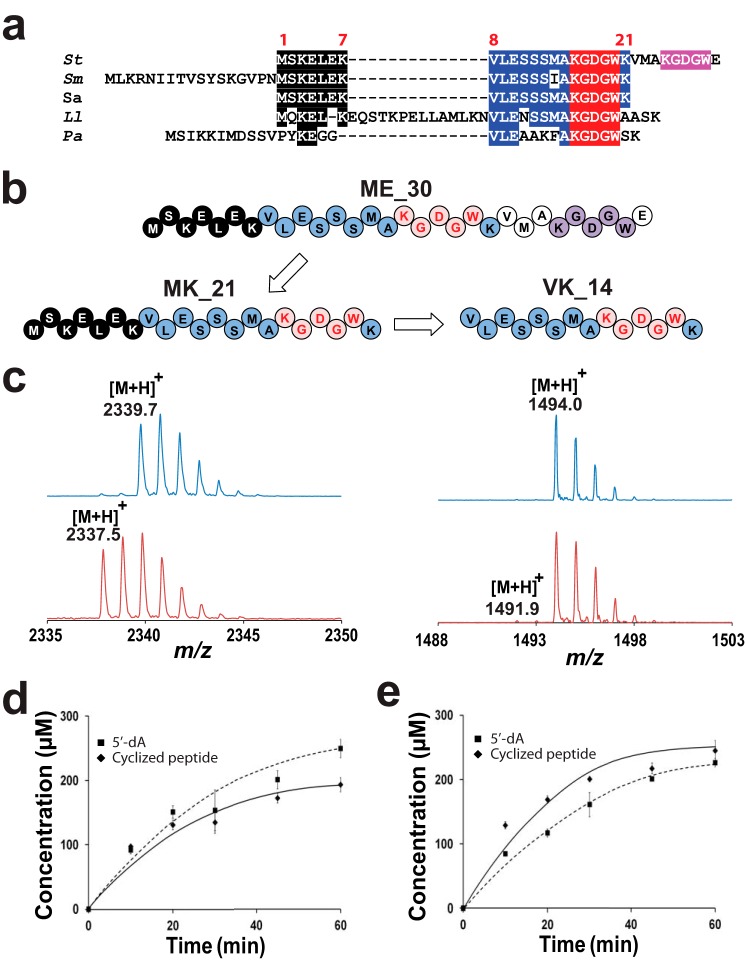
**Minimal substrate for KW_cyclase.** *a*, sequence alignment of ME_30 peptide homologs found in various bacteria. The putative peptide leader sequence is highlighted in *black*. The conserved residues are highlighted in *blue*, whereas the residues involved in the cyclic KGDGW peptide are highlighted in *red*. The second KGDGW motif, found in *S. thermophilus* is highlighted in *purple. St*, *S. thermophilus*; *Sm*, *S. mitis*; *Sa*, *S. agalactiae*; *Ll*, *L. lactis*; *Pa*, *P. aeruginosa. b*, sequences of the ME_30, MK_21, and VK_14 peptides. *c*, MALDI-TOF MS analysis of MK_21 (*left panel*) and VK_14 (*right panel*) peptides before (*top traces*) or after (*bottom traces*) incubation with KW_cyclase (see below for experimental conditions). *d*, kinetic analysis of the reaction catalyzed by KW_cyclase in the presence of the ME_30 peptide. The data represent the mean ± S.D. of three independent reactions. See below for experimental conditions. *e*, kinetic analysis of the reaction catalyzed by KW_cyclase in the presence of the MK_21 peptide. Reactions were performed by incubating the reconstituted KW_cyclase (68 μm) with peptide substrate (1 mm), SAM (1 mm), DTT (3 mm), and sodium dithionite (2 mm) under anaerobic conditions. The data represent the mean ± S.D. of three independent reactions.

A search in the upstream region of these radical SAM enzymes led to the identification of small ORFs coding for putative peptides containing the KGDGW motif (Fig. 3*a*), whereas, in the downstream regions, we identified genes coding for a putative protease and ABC transporter, similar to the *S. thermophilus* operon. Sequence alignment between these putative peptides led to the identification of a minimal consensus peptide containing residues 1–21 with a core region encompassing residues 8–21. Previous attempts to assay the KW_cyclase with peptides shorter than the ME_30 peptide failed to identify suitable substrates, suggesting that the whole sequence is important for interaction or activity (8).

To verify this hypothesis, we synthesized a peptide containing the strictly conserved residues 1–21 (peptide MK_21) (Fig. 3*b*). Surprisingly, this sequence, which corresponds to the sequence identified notably in the human pathogen *S. agalactiae*, proved to be an efficient enzyme substrate (Fig. 3, *b* and *c*). Indeed, with this shorter substrate ([M+H]^+^, 2339.7), a new product ([M+H]^+^, 2337.5) consistent with cyclization activity was formed. To ensure the nature of the product formed, we characterized this peptide by NMR spectroscopy (supplemental Figs. S3–S7). After trypsin digestion, the peptide containing residues 8–21, including the KGDGW motif, was purified by HPLC. The ^1^H and ^13^C NMR signals of the constituent amino acids were assigned using a combination of 2D NMR experiments, including COSY, TOCSY, NOESY, HSQC, HMBC, and HSQC-TOCSY (supplemental Figs. S3–S7 and Table S4). The amino acid sequence of the peptide was confirmed by sequential assignment using the *d*αN(i, i+1) NOE connectivities. Comparison of the TOCSY and HSQC spectra of the linear and cyclic peptide showed that the spin system of Lys-9 (corresponding to Lys-16 in the full-length peptide) was strongly modified. The H_α_ proton of Lys-9 was shifted downfield by 0.4 ppm and appeared as a doublet instead of a triplet, indicating abstraction of a proton from the carbon-β. The ^3^J_αβ_ value of 11 Hz suggested a restricted rotation around the Cα-Cβ bond with an HCCH torsion angle of around 180°. In the COSY spectrum, H_α_(Lys-9) showed a cross-peak to a signal at 3.5 ppm assigned as H_β_(Lys-9), thus deshielded by approximately 1.8 ppm compared with H_β_(Lys-14) (corresponding to Lys-21 in the full-length peptide). C_β_(Lys-9) was also deshielded by 10 ppm compared with C_β_(Lys-14), and the multiplicity-edited HSQC revealed that C_β_(Lys-9) was a methine and not a methylene carbon, demonstrating abstraction of a proton at this position. The ^1^H NMR spectra and the HSQC spectra of the aromatic region of tryptophan showed only four signals corresponding to four CH groups instead of the five normally observed for unsubstituted tryptophan. This demonstrates abstraction of one proton on one of the carbons of the benzene ring. Analysis of the spin system showed that the C6 proton on the benzene ring appeared as a doublet (instead of a triplet in tryptophan), and this, together with the HMBC spectrum, showed that abstraction occurred at C7 on the six-membered ring. Cyclization was confirmed based on the observation of a diagnostic NOE between H_α_ of Lys-9 and C6 of Trp and between NH-1 of the tryptophan ring at 10.42 ppm and H_β_ of Lys-9 at 3.57 ppm. Thus, NMR analysis unambiguously established that, using this shorter substrate, the expected C_β_-C7 bond between lysine and tryptophan was formed as found in the peptide naturally produced by *S. thermophilus* (8).

It has been shown recently, in the case of AlbA (6) or PoyC (9), two radical SAM enzymes catalyzing peptide modification, that the leader peptide was dispensable for activity. To test the importance of the leader sequence, we synthesized a novel substrate deprived of the first seven amino acids, which define a conserved sequence in the N-terminal region (Fig. 3*a*). This novel peptide containing residues 8–21 (peptide VK_14, [M+H]^+^, 1494.0) proved to be an enzyme substrate, albeit with a very low amount of cyclic peptide produced (<1%; [M+H]^+^, 1491.9; Fig. 3*c*). The MK_21 peptide thus appears to be the minimal substrate for efficient enzyme activity. This result supports a strong dependence of the KW_cyclase to the leader sequence. Kinetic experiments, performed in the presence of the ME_30 or MK_21 peptide, showed that the production of cyclized peptide and 5′-dA is faster in the presence of the latter peptide, with a *k*_cat_ of 0.09 ± 0.005 min^−1^ and 0.14 ± 0.01 min^−1^, respectively (Fig. 3, *d* and *e*). Interestingly, with both substrates, we measured a good correlation between the productions of 5′-dA and cyclized peptides, supporting that 1 mole of SAM is consumed per catalytic cycle.

### Iron-sulfur clusters of the KW_cyclase

**Figure 4.**
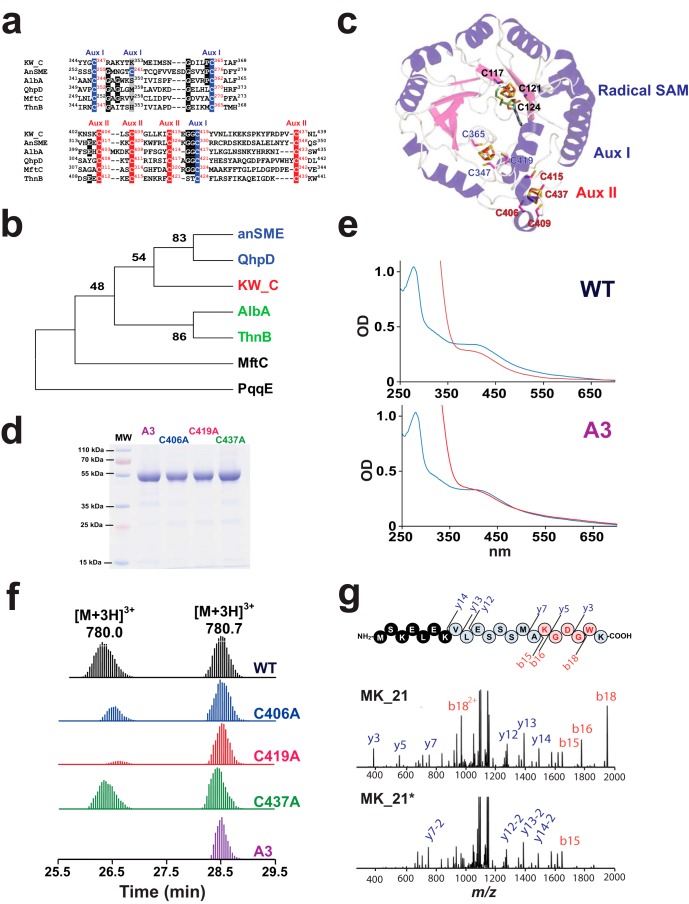
**Investigation of the [4Fe-4S] clusters present in KW_cyclase.** *a*, sequence alignment of the conserved cysteine residues present in KW_cyclase (*KW*_*C*), anSME, AlbA, PqqE, and MftC. The cysteine residues involved in the coordination of auxiliary cluster I (*Aux I*) and II (*Aux II*), according to the anSME structure, are highlighted in *blue* and *red*, respectively. Residues conserved among at least three sequences are highlighted in *black*. Amino acids occupying the position of Cys-261 (in anSME) are highlighted in *gray. b*, molecular phylogenetic analysis of representative SPASM domain radical SAM enzymes: KW_cyclase, anSME, AlbA, PqqE, and MftC. The evolutionary history was inferred by using the maximum likelihood method. The percentage of replicate trees in which the associated taxa clustered together in the bootstrap test is indicated next to the branches (1000 replicates). The SPASM domain radical SAM enzymes catalyzing protein modifications are highlighted in *blue*. SPASM domain radical SAM enzymes catalyzing thioether bond formation on peptides are highlighted in *green. c*, structural model of the KW_cyclase. SAM is depicted in *green* and colored by atom elements. Cysteine residues predicted to be involved in the coordination of the radical SAM [4Fe-4S] (Cys-117, Cys-121, and Cys-124) and the coordination of the auxiliary cluster I (Cys-347, Cys-365, and Cys-419) and auxiliary cluster II (Cys-406, Cys-409, Cys-415, and Cys-437) are indicated. *d*, gel electrophoresis analysis (SDS-PAGE, 12.5%) of the purified A3, C437A, C419A, and C406A mutants. *MW*, molecular weight. *e*, UV-visible spectrum of the wild-type (10 μm, *top traces*) and the A3 mutant (10 μm, *bottom trace*) before (*blue traces*) and after (*red traces*) 20-min incubation with sodium dithionite. *OD*, optical density. *f*, LC/MS analysis of the peptide MK_21 incubated with the WT or the A3, C437A, C419A, or C406A mutants. Reactions were performed by incubating the respective proteins (50 μm) after anaerobic reconstitution with the MK_21 peptide substrate (1 mm), SAM (1 mm), DTT (3 mm), and sodium dithionite (2 mm) for 2 h. *g*, LC/MS-MS analysis of the MK_21 peptide and the cyclic MK_21* peptide produced by the (WT) KW_cyclase. The characteristic ions are indicated. Similar fragmentation patterns were obtained for the C437A, C419A, and C406A mutants.

As described above, iron and sulfur quantifications support that the KW_cyclase contains up to three [4Fe-4S] clusters, contrary to previous reports. Among the 13 cysteine residues present in the sequence of the enzyme, Cys-409 and Cys-415 have been mutated previously and proposed, by homology with anSME, to be involved in the coordination of a SPASM domain [4Fe-4S] cluster. The structural analysis of anSME (20) has revealed that four cysteine residues, Cys-317, Cys-320, Cys-326, and Cys-348, coordinate one [4Fe-4S] cluster (called auxiliary II), with the residues Cys-320 and Cys-326 being the equivalent of Cys-409 and Cys-415 in the KW_cyclase (11, 15, 19–21) (Fig. 4*a*). However, in anSME a second [4Fe-4S] cluster was also identified, buried within the enzyme structure and coordinated by four additional cysteine residues: Cys-255, Cys-261, Cys-276, and Cys-330. This last cluster, close to the radical SAM cluster, was labeled auxiliary I.

Sequence alignment of the KW_cyclase with five biochemically characterized SPASM domain radical SAM enzymes (anSME, AlbA, QhpD, MftC, and ThnB), showed clear conservation of the four cysteine residues involved in the coordination of the auxiliary cluster II (Fig. 4*a*). Strikingly, all the SPASM domain radical SAM enzymes also possess three conserved cysteine residues of the four involved in the coordination of the auxiliary cluster I in anSME (Fig. 4*a*). In addition, despite these enzymes having no significant homology (identity ranging between 16–26%), it is always the same cysteine residue (Cys-261 in anSME) that is substituted. Of note, this residue is often replaced by residues known to be involved in the coordination of [4Fe-4S] clusters such as serine, histidine, or lysine residues (26, 27). This suggests a possible coordination of auxiliary cluster I by three cysteine residues and a non-cysteinyl ligand (27) in these enzymes. Phylogenetic analysis revealed that the KW_cyclase clustered with anSME and three enzymes catalyzing thioether bond formation, including AlbA, whose SPASM domain has been shown recently to contain two [4Fe-4S] clusters (Fig. 4*b*).

To probe the function of these cysteine residues, we built a structural model for the KW_cyclase (Fig. 4*c*) using the I-Tasser server (28). The model predicted the coordination of auxiliary cluster II by Cys-406, Cys-409, Cys-415, and Cys-437. This model also disclosed that Cys-347, Cys-365, and Cys-419 are perfectly positioned to coordinate auxiliary cluster I (Fig. 4*c*), as suggested by sequence alignment (Fig. 4*a*). To probe for the existence of this cluster, we constructed three Cys-to-Ala single mutants: C406A, C419A, and C437A. We also constructed a triple mutant (A3) in which the residues Cys-117, Cys-121, and Cys-124, predicted to coordinate the radical SAM [4Fe-4S] cluster, were mutated to alanine. All mutants were successfully purified (Fig. 4*d*), and, after anaerobic reconstitution, their UV-visible spectra were recorded (supplemental Fig. S8). The three single Cys-to-Ala mutants had UV spectra and iron-sulfur contents (85% ± 17%) comparable with the wild-type enzyme. The A3 mutant contained 11.6 ± 0.2 mol of iron and 14.0 ± 0.3 mol of sulfide per mole of protein, suggesting the presence of more than one [4Fe-4S] cluster.

Upon addition of sodium dithionite, the UV-visible absorption spectrum of the wild-type enzyme showed a decrease in absorbance in the 420- to 600-nm region (∼20%), consistent with the presence of one redox-active [4Fe-4S] cluster (Fig. 4*e*). In contrast, under similar conditions, the UV-visible spectrum of the A3 mutant exhibited no significant change, indicating that it contains no [4Fe-4S] cluster reducible under these conditions (Fig. 4*e*).

We further assayed the activity of the four mutants against the MK_21 peptide. As expected, the A3 mutant was unable to cleave SAM and did not lead to formation of any peptide product (Fig. 4*f*), demonstrating the role of Cys-117, Cys-121, and Cys-124 in the coordination of the radical SAM cluster. In contrast, the three single Cys-to-Ala mutants were able to reductively cleave SAM and to produce cyclic peptide, albeit at various levels (Figs. 4, *f* and *g*, and supplemental Fig. S9). The C437A mutant exhibited activity similar to the WT enzyme, whereas the C406A (mutated in the Auxiliary cluster II) and C419A (mutated in the Auxiliary cluster I) mutants exhibited very low levels of activity. Careful HPLC and LC/MS analyses failed to evidence the formation of any additional peptide products that could have accounted for reaction intermediates.

Collectively, these results show that, in addition to Cys-409 and Cys-415, Cys-406, Cys-419, and Cys-437 are not only conserved across SPASM domain radical SAM enzymes but that they are also important for enzyme activity, supporting their involvement in the coordination of two SPASM domain [4Fe-4S] clusters.

## Discussion

SPASM domain radical SAM enzymes are emerging as a major class within the radical SAM enzyme superfamily. Until now, all SPASM domain radical SAM enzymes identified have been shown to be involved in protein or peptide post-translation modifications (Fig. 1) (7, 14, 15, 21). They have been reported to catalyze C_β_ (*e.g.* anSME (14) and KW_cyclase (8)) and C_α_ H atom abstraction (*e.g.* AlbA (6)), and they are predicted to catalyze Cγ H atom abstraction (*e.g.* QhpD (29) and PqqE). Interestingly, if they all catalyze the loss of two H atoms, these enzymes lead to different transformations, including amino acid oxidation and thioether and carbon-carbon bond formations (Fig. 1).

The initial step of these reactions is the reductive cleavage of SAM and the radical abstraction of an amino acid H atom. However, the next steps are not well understood. For anSME and AlbA, following H atom abstraction, the radical intermediate is further oxidized to a thioaldehyde (11, 14, 15) or an *N*-acyliminium ion (6) reaction intermediate, respectively. In the case of the KW_cyclase and PqqE, the most logical pathway implies radical addition to an aromatic residue, leading to the formation of a carbon-carbon bond.

Our study supports that the KW_cyclase is strictly specific for the Lys and Trp residues and cannot perform the cyclization reaction when these residues are mutated. We also show that this enzyme is not active on a peptide lacking the first eight amino acid residues, demonstrating that it requires an intact leader peptide for activity in contrast to other SPASM domain radical SAM enzymes (6, 14, 15, 30). Interestingly, in the ME_30 peptide (Fig. 2*c*), the KGDGW motif is duplicated, but this second motif is never modified, as demonstrated by the absence of activity on the peptide variants (Fig. 2*f*). The function of the leader peptide is thus likely to correctly position the peptide within the enzyme active site.

We also proved that the core sequence, composed of 21 residues, is an efficient substrate. Interestingly, this sequence is present in the human pathogen *S. agalactiae*, suggesting that this peptide might be produced by this bacterium and likely other bacteria, including streptococci, lactococci, and even the Gram-negative bacterium *P. putida.*

Previous reports have indicated that mutation of two conserved cysteine residues, predicted to coordinate auxiliary cluster II, hinders enzyme activity. We show here that single mutation of cysteine residues involved in the coordination of auxiliary clusters II and I only reduces the activity of the enzyme. Indeed, all enzyme variants produced cyclic peptides *in vitro*, except the enzyme lacking the radical SAM cluster (mutant A3). Intriguingly, among the SPASM domain radical SAM enzymes characterized to date, only anSME possesses eight conserved cysteine residues in its SPASM domain (11, 20), which are involved in the coordination of two [4Fe-4S] clusters. In the recently discovered SPASM domain enzymes (*i.e.* AlbA, ThnB, QhpD, MtfC, and KW_cyclase), and possibly in the most distantly related PqqE enzyme (11), seven cysteine residues are strictly conserved with anSME (Fig. 4*a*). Interestingly, all of these SPASM domain radical SAM enzymes lack the same cysteine residue, which corresponds to Cys-261, involved in the coordination of auxiliary cluster I in anSME (11, 19). It is very unlikely that all of these enzymes have conserved the auxiliary cluster II and lost the auxiliary cluster I, which is the most buried one, in close proximity with the radical SAM cluster and likely to interact with the substrates of the respective enzymes (either directly or indirectly). Conversely, it is striking that three of four cysteine residues are conserved in these enzymes despite sharing no significant sequence homologies. Our data support that these novel SPASM domain radical SAM enzymes likely coordinate the auxiliary cluster I using three cysteine residues and a non-cysteine ligand. Recent examples of such a type of coordination have been found in the radical SAM enzyme lipoyl synthase (26, 31) and in several other iron-sulfur proteins.

These non-cysteine ligands could be used by SPASM domain radical SAM enzymes to finely tune the redox properties of the [4Fe-4S] clusters, as shown for fumarate reductase (32) or glutaredoxins (33). However, after AlbA (6) and now the KW_cyclase, it is likely that a growing number of SPASM domain radical SAM enzymes will exhibit a similar type of [4Fe-4S] cluster coordination. Further studies will undoubtedly explain why nature evolved such a sophisticated control of iron-sulfur clusters and their reactivity.

## Experimental procedures

### Cloning, expression, and enzyme purification

The gene *ster*_*1356* coding for the KW_cyclase from *S. thermophilus* LMD-9 was synthesized by Life Technologies and expressed as a Strep-tag fusion protein using a pASK plasmid. The gene construct was verified by sequencing. The construct was transformed in *E. coli* BL21 (DE3) for protein expression. Transformed *E. coli* was grown in Luria-Bertani medium using the following conditions. The growth medium was supplemented with ampicillin (100 μg ml^−1^), and bacterial growth was performed at 37 °C until *A*_600 nm_ reached 0.6. The expression of the Ster_1356 protein was induced by adding anhydrotetracycline (200 nm final concentration) to the cells, and protein expression was performed during 18 h at 20 °C. Cells were harvested by centrifugation and resuspended in Tris-HCl buffer (pH 7.5) (buffer A: 50 mm Tris and 300 mm KCl). Cells were disrupted by sonication in the presence of 1% (v/v) Triton X-100 and 1‰ (v/v) 2-mercaptoethanol, and the supernatant was collected after centrifugation at 45,000 × *g* during 1 h. Protein purification was done on a Strep-Tactin column equilibrated with buffer A, and the protein was eluted with 1 mm desthiobiotin in the presence of 3 mm dithiothreitol in buffer A. The protein was concentrated and stored at −80 °C. The genes encoding the protein variants C406A, C419A, C437A, and C117A/C121A/C124A were synthesized by Life Technologies. These genes were expressed, and the proteins were purified similarly as the wild-type enzyme.

### Enzymatic assays

The peptides used in this study were ordered from Proteogenix with a purity of >98%. For [Fe-S] cluster reconstitution, proteins were incubated for 12 h at 6 °C in the presence of 3 mm DTT with a 10-fold molar excess of (NH4)_2_Fe(SO4)_2_ (Sigma-Aldrich) and Na_2_S (Sigma-Aldrich). The proteins were desalted on a PD-10 column and concentrated.

Enzymatic assays contained 50 μm reconstituted protein, 1 mm SAM, 3 mm DTT, 1 mm peptide, and 2 mm sodium dithionite unless otherwise indicated. Samples were incubated at 20 °C under anaerobic and reducing conditions. Kinetic analyses were performed in triplicate.

### HPLC analysis

HPLC analysis was carried out on an Agilent 1200 series Infinity chromatographic system. Samples were diluted 1:10 in H_2_O containing 0.1% (v/v) TFA. A reverse-phase column (LiChroCART RP-18e, 5 μm) was equilibrated with 100% solvent A (H_2_O, 0.1% (v/v) TFA). The gradient of 1.2%/min was applied using solvent B (80% (v/v) CH_3_CN, 0.1% TFA) and a flow rate of 1 ml/min.

### LC/MS-MS analysis

Peptide mass analyses were realized on a MALDI-TOF mass spectrometer (Voyager DEstr, Applied Biosystems) in reflector mode with α-cyano-4-hydroxycinnamic acid as a matrix. Peptide fragmentation analyses were realized by coupling liquid chromatography to mass spectrometry using an Ultimate 3000 LC system (Dionex) connected to an LTQ or Qexactive mass spectrometer (Thermo Scientific) in positive mode with a nanoelectrospray ion source. The samples were diluted 100 times in formic acid 0.1% (v/v). 1 μl of sample was directly injected onto Pepmap100 C18 (0.075 × 15 cm, 100 Å, 3 μm) and eluted by a linear gradient of 2% (v/v)/min of mobile phase B (80% (v/v) CH_3_CN, 20% H_2_O, 0.1% formic acid) during 25 min at a flow rate of 300 nl min^−1^. The doubly charged ion corresponding to peptides MK21, MK21*, VK14, and VK14* were selected for fragmentation by collision-induced dissociation or higher-energy collisional dissociation in the linear ion trap or in the Ctrap with a normalized collision energy fixed to 35%.

### NMR spectroscopy

The freeze-dried linear and cyclic 14-amino acid samples were dissolved in 120 μl of D_2_O (99.96%) and transferred into 3-mm NMR sample tubes. The NMR spectra were recorded on a Bruker AVANCE^TM^ III 600-MHz spectrometer using a 5-mm ^1^H/^13^C/^15^N/^31^P cryoprobe equipped with a z gradient. The optimum temperature at which there was no overlap of the water signal with signals from the amino acids was 278 K. The residual HOD signal was used as reference (δ_H_ 4.9955 at 278 K) for ^1^H chemical shifts. The assignments of ^1^H and ^13^C resonances were obtained from ^1^H-^1^H COSY, TOCSY, and NOESY and from ^1^H-^13^C multiplicity-edited HSQC and HMBC experiments from the Bruker pulse sequence library. A mixing time of 120 ms was used for TOCSY and 100 and 500 ms for NOESY experiments. For assignment of NH resonances and establishment of the peptide sequence, the cyclic peptide sample was dissolved in 60% D_2_O/40% H_2_O. The TOCSY and NOESY spectra were obtained at 278 K as for D_2_O solutions.

### Protein structure prediction of KW_cyclase

The structural model was built using I-Tasser (28). The best identified structural analog in the PDB was anSME (PDB code 4K36). We generated a model of KW_cyclase encompassing residues 103–337 and anSME (PDB code 4K36) as a template.

### Iron and sulfide titration

Determination of the iron and sulfide content of the protein samples was performed as follows. For iron titration, 100 μl of 5 μm enzyme was mixed with 100 μl of HCl 1% (v/v) and incubated at 80 °C for 10 min. Tubes were allowed to cool down at room temperature prior to sequential addition of 500 μl of 7.5% ammonium acetate, 100 μl of 4% (w/v) ascorbic acid, and 100 μl of 2.5% (w/v) SDS. Iron chelation was achieved by adding 100 μl of 1.5% Ferene (w/v), followed by 10-min centrifugation at 13,000 × *g*. The (Ferene)_3_-Fe(II) complex absorbance was recorded at 593 nm (ε = 33.5 mm^−1^ cm^−1^).

For sulfide titration, 200 μl of 5 μm enzyme was mixed with 600 μl of 1% (w/v) zinc acetate and 50 μl of 7% (v/v) NaOH. Simultaneous addition of 0.1% (v/v) *N,N'*-dimethyl-*p*-phenylene-diamine (DMPD) and 10 mm FeCl_3_ was performed, and, after centrifugation, methylene blue absorbance was recorded at 670 nm (ε = 27.4 mm^−1^ cm^−1^).

## Author contributions

A. B., L. D., A. G., P. R., X. K., C. S., and O. B. performed the experiments and analyzed the data. A. B. and O. B. directed the study, analyzed the data, and wrote the manuscript. All authors approved the final version of the manuscript.

## Supplementary Material

Supplemental Data

## Footnotes

3 The abbreviations used are:

RiPP: ribosomally synthesized and post-translationally modified peptide
SPASM: subilitosin, PQQ, anaerobic sulfatases, and mycofactocin
SAM: *S*-adenosylmethionine
TOCSY: total correlation spectroscopy
HSQC: heteronuclear single quantum coherence
HMBC: heteronuclear multiple bond correlation.
