# Supplementary material for: Insights into the catalysis of a lysine-tryptophan bond in bacterial peptides by a SPASM domain radical S-adenosylmethionine (SAM) peptide cyclase
Source: J Biol Chem. 2017 May 5;292(26):10835–44. doi: 10.1074/jbc.M117.783464 (PMC5491770; doi:10.1074/jbc.M117.783464)
Supplement: Supplemental Data [file supp_292_26_10835__index.html]

Insights into Catalysis of Lysine-Tryptophan Bond in Bacterial Peptides by a SPASM-Domain Radical SAM Peptide Cyclase — Insights into the catalysis of a lysine-tryptophan bond in bacterial peptides by a SPASM domain radical S-adenosylmethionine (SAM) peptide cyclase — Catalysis of C–C bond by a SPASM domain radical SAM enzyme — Supplemental Data 

# Insights into the catalysis of a lysine-tryptophan bond in bacterial peptides by a SPASM domain radical *S*-adenosylmethionine (SAM) peptide cyclase

## Supplemental Data

- Supplemental data (.pdf, 1.2 MB) - Supplemental data
