# Supplementary material for: Insights into the catalysis of a lysine-tryptophan bond in bacterial peptides by a SPASM domain radical S-adenosylmethionine (SAM) peptide cyclase
Source: J Biol Chem. 2017 May 5;292(26):10835–44. doi: 10.1074/jbc.M117.783464 (PMC5491770; doi:10.1074/jbc.M117.783464)
Supplement: Supplemental Data [file 10.1074_M117.783464_jbc.M117.783464-1.pdf]

Supporting information for:

Insights into Catalysis of Lysine-Tryptophan Bond in Bacterial Peptides by a SPASM Domain Radical SAM Peptide Cyclase

Alhosna Benjdia*[1], Laure Decamps[1], Alain Guillot[1], Xavier Kubiak[1], Pauline Ruffié[1], Sandström Corine[2] & Olivier Berteau[1]*

[1] Micalis Institute, ChemSyBio, INRA, AgroParisTech, Université Paris-Saclay, 78350 Jouy-en-Josas, France

[2] Department of Molecular Sciences, Uppsala BioCenter Swedish University of Agricultural Sciences, P.O. Box 7015, Uppsala SE-750-07, Sweden.

To whom correspondence should be addressed: Olivier Berteau, INRA, Institut Micalis (UMR 1319), ChemSyBio, F-78350 Jouy-en-Josas, France. Tel. :+33(0)1 34 65 23 08 Fax : +33(0)1 34 65 24 62, E-mail : Alhosna Benjdia : Alhosna.Benjdia@ inra.fr Olivier Berteau : Olivier.Berteau@inra.fr

Figure S1 3
Figure S2 4
Figure S3 5
Figure S4 5
Figure S5 6
Figure S6 6
Figure S7 7
Figure S8 8
Figure S9 9
Table S1 - Fragmentation pattern of the tryptic peptide 8-21 10
Table S2 - Fragmentation pattern of the tryptic peptide 8-21* 10
Table S3 – Mass of the tryptic peptide obtained from ME_30 11
Table S4 – ^{1}H and ^{13}C NMR chemical shifts of the cyclic peptide 12

Figure S1

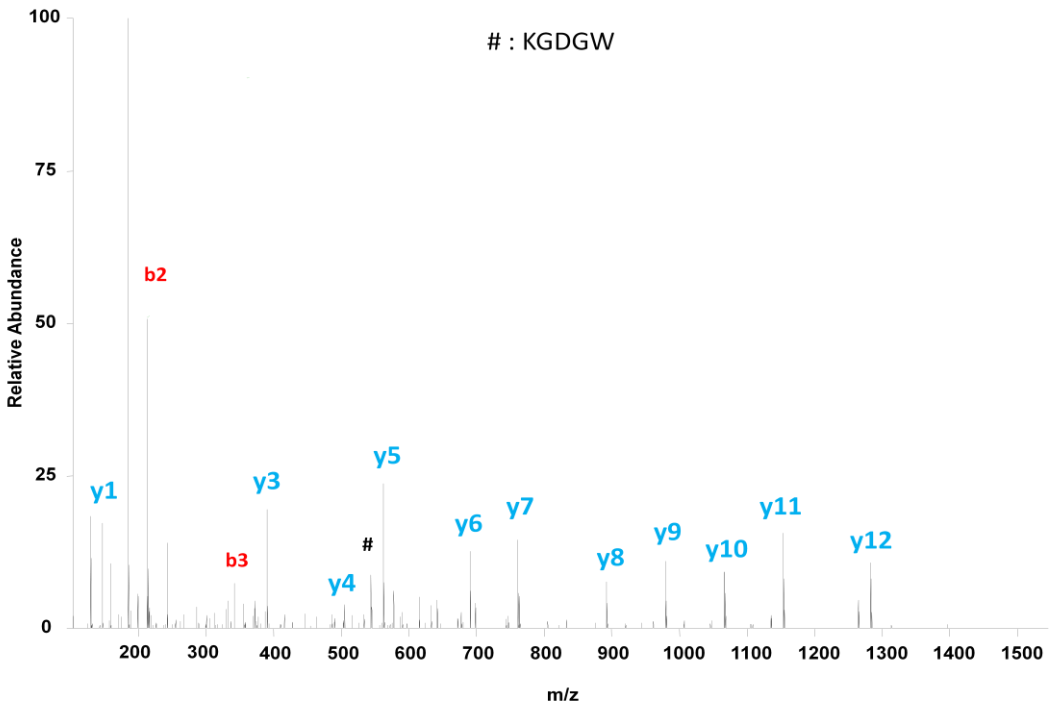

Figure S1 – LC-MS analysis of the tryptic peptide encompassing the KGDGW motif from the ME_30 peptide.

Figure S2

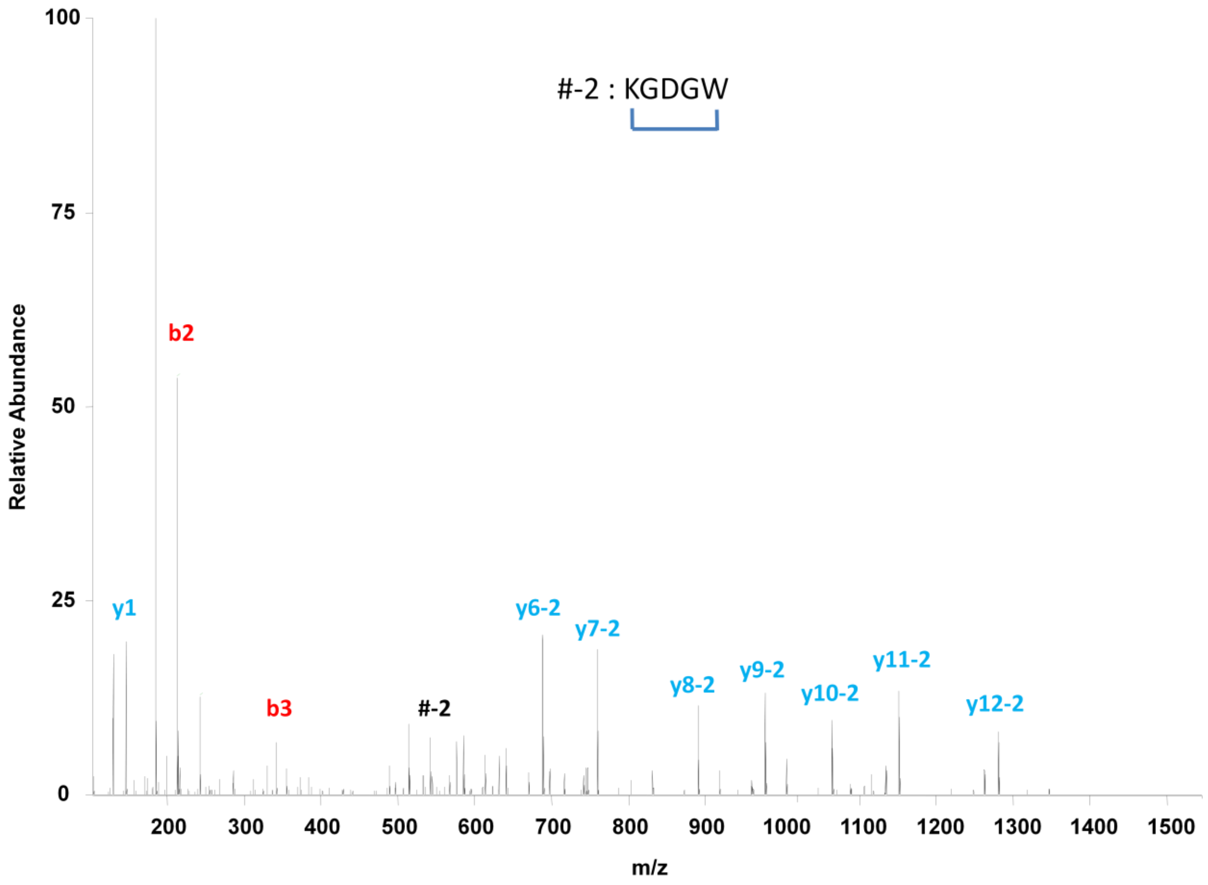

Figure S2 – LC-MS analysis of the tryptic peptide containing the KGDGW motif after incubation of the ME_30 peptide with the reconsituted KW_cyclase under anaerobic conditions.

Figure S3

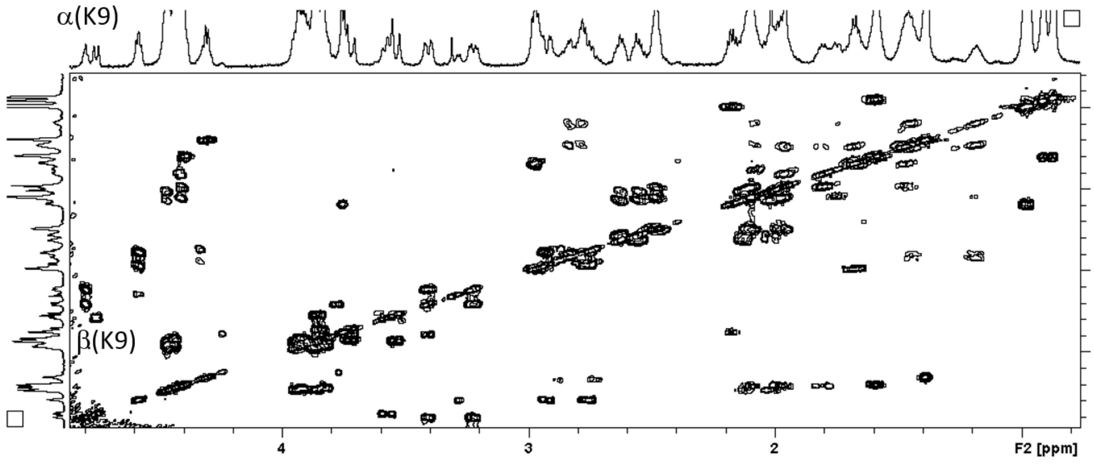

^{1}H-^{1}H COSY NMR-spectrum of the 14-mer cyclic peptide showing the downfield shift of Hα and especially of Hβ of K9

Figure S4

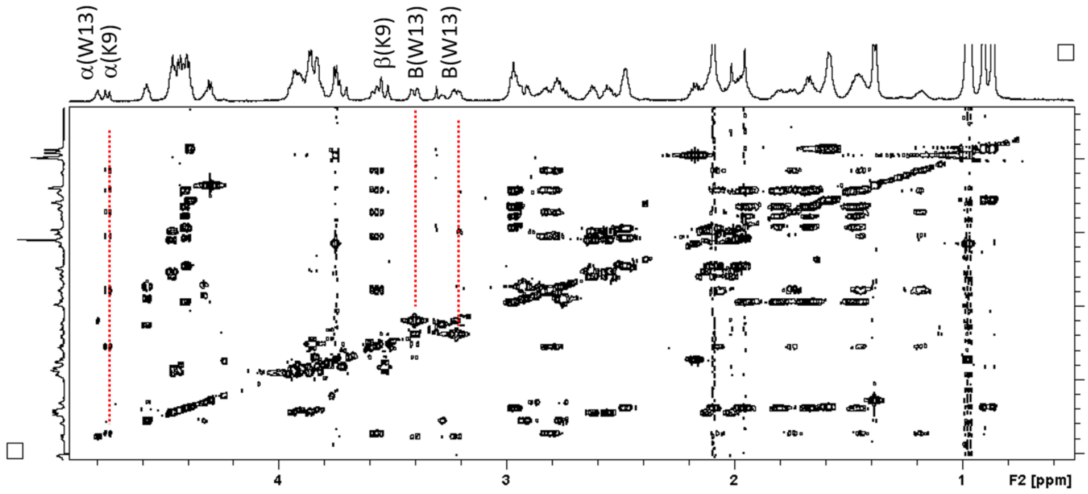

^{1}H-^{1}H TOCSY NMR-spectrum of the 14-mer cyclic peptide showing the spin system of K9 and the two well separated Hβ (W13) signals.

Figure S5

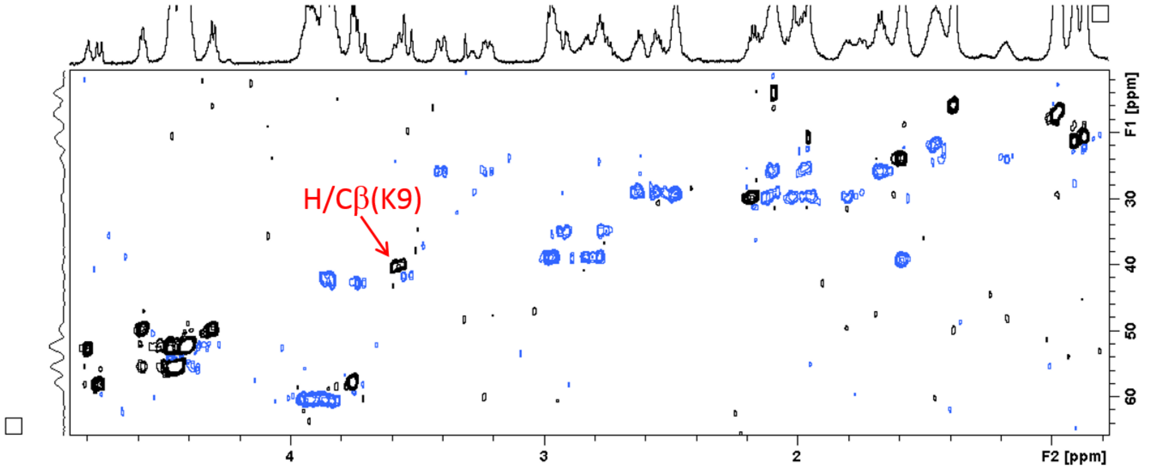

^{1}H-^{13}C Multiplicity-edited HSQC NMR-spectrum of the 14-mer cyclic peptide. The CH and CH_3 are in blacks while the CH_2 are in blue.

Figure S6

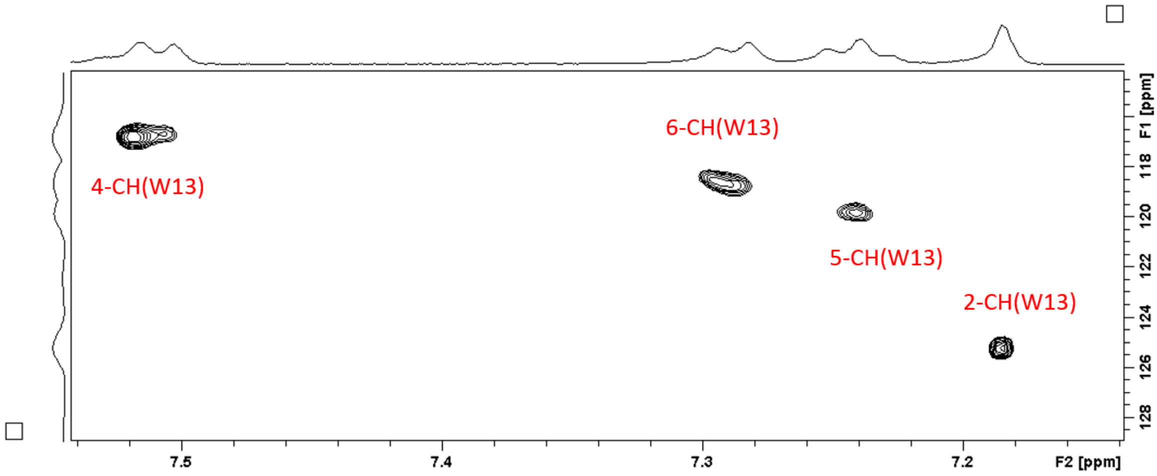

^{1}H-^{13}C Multiplicity-edited HSQC NMR-spectrum of the 14-mer cyclic peptide showing the aromatic region of tryptophane.

Figure S7

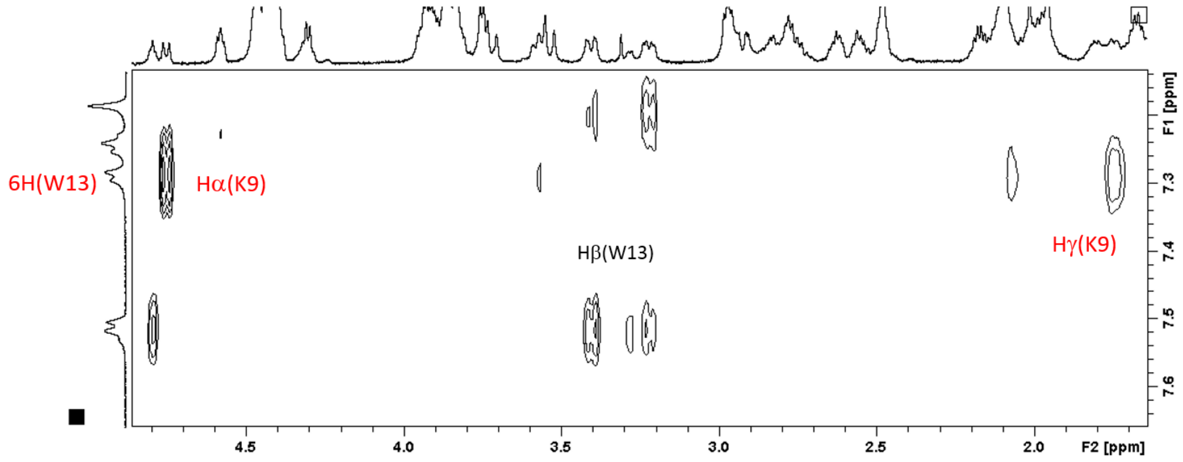

^{1}H-^{1}H NOESY NMR-spectrum of the 14-mer cyclic peptide showing the diagnostic NOE between Hα of K9 and H6 of W13.

Figure S8

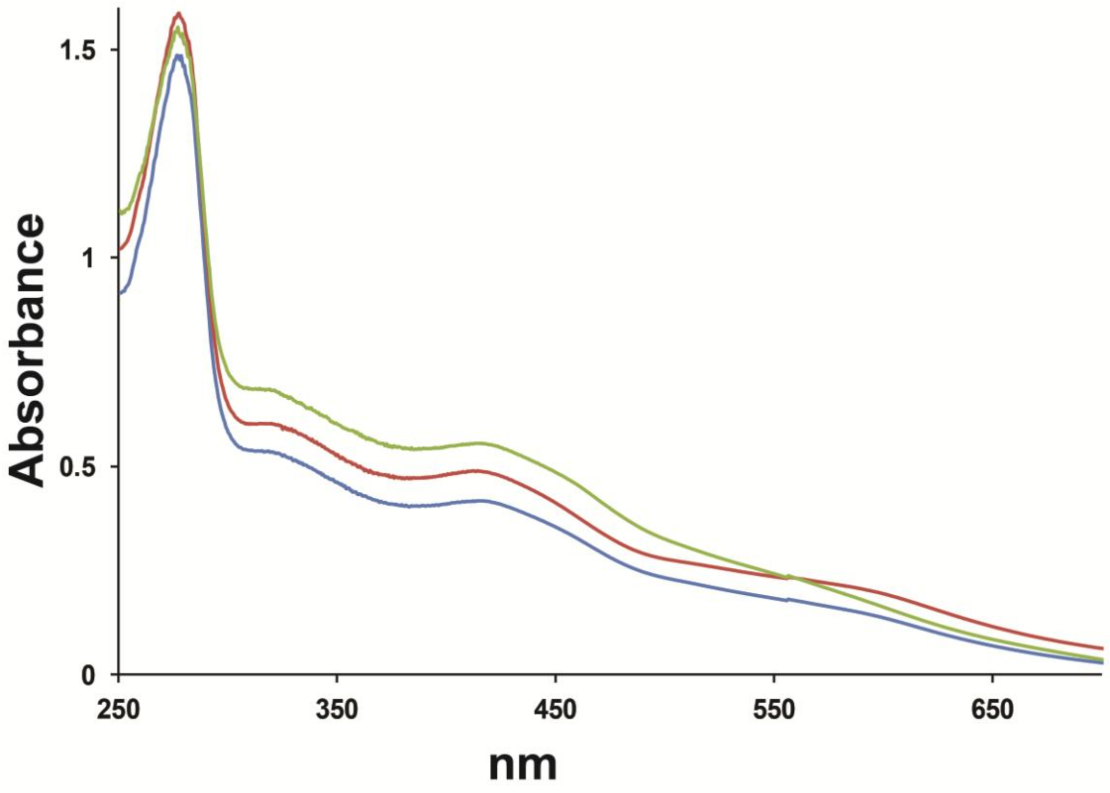

Figure S8 – UV-visible spectrum of the KW_cyclase mutants C406A (blue trace), C419A (red trace) and C437A) (green trace).

All mutants were reconstituted under anaerobic conditions prior spectroscopy analysis.

Figure S9

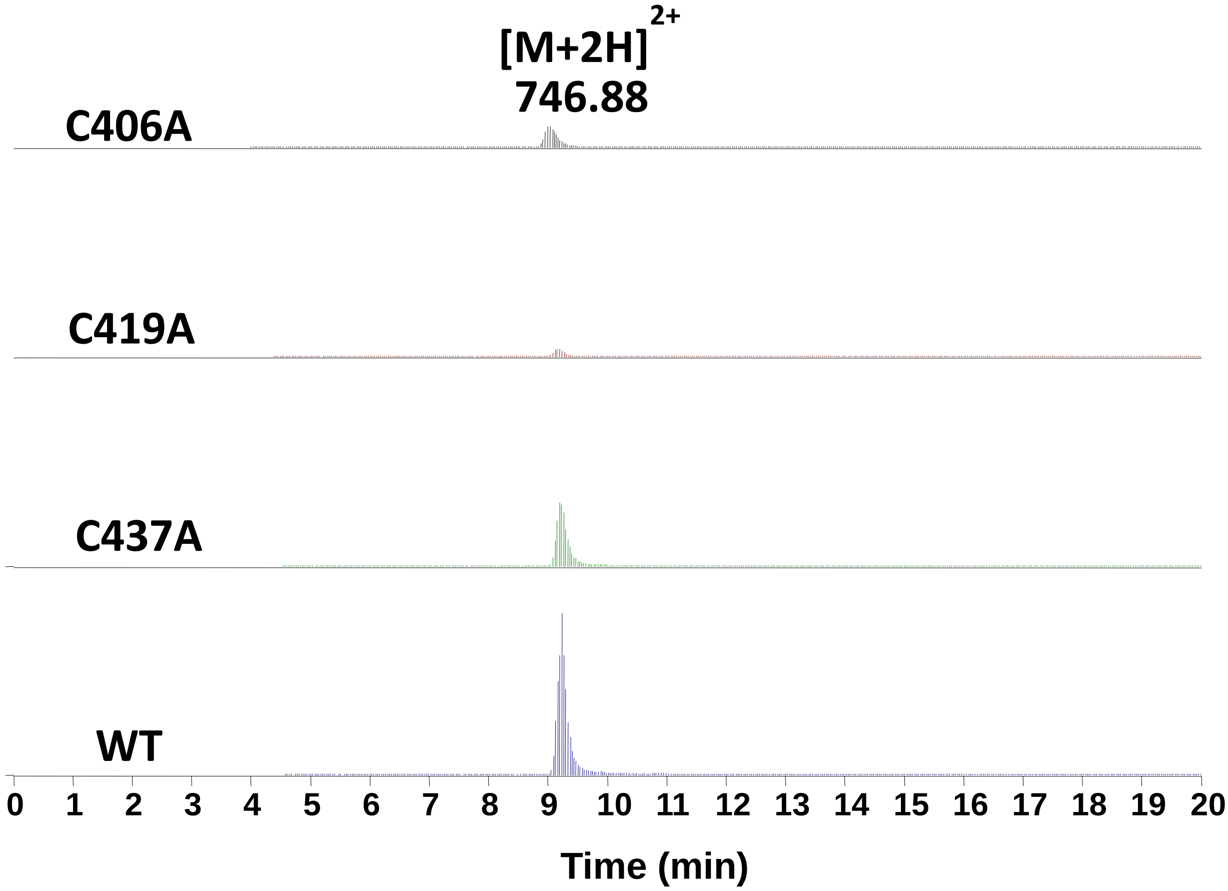

Figure S9 – Normalized extracted ion current corresponding to the VK_14 peptide fragment containing the cyclic motif KGDGW. Tryptic peptide fragment was obtained after incubation of the MK_21 peptide with the wild type KW_cyclase (WT) or the corresponding mutants: C406A, C419A and C437A.

Table S1 - Fragmentation pattern of the tryptic peptide 8-21

Sequence		b	y	
V	1	100.07628	1494.72630	14
L	2	213.16035	1395.65788	13
E	3	342.20294	1282.57382	12
S	4	429.23497	1153.53123	11
S	5	516.26700	1066.49920	10
S	6	603.29902	979.46717	9
M	7	734.33951	892.43514	8
A	8	805.37662	761.39466	7
K	9	933.47159	690.35754	6
G	10	990.49305	562.26258	5
D	11	1105.51999	505.24112	4
G	12	1162.54146	390.21417	3
W	13	1348.62077	333.19271	2
K	14	1476.71573	147.11340	1

Table S2 - Fragmentation pattern of the tryptic peptide 8-21*

Sequence		b	y	
V	1	100.07628	1492.71065	14
L	2	213.16035	1393.64223	13
E	3	342.20294	1280.55817	12
S	4	429.23497	1151.51558	11
S	5	516.26700	1064.48355	10
S	6	603.29902	977.45152	9
M	7	734.33951	890.41949	8
A	8	805.37662	759.37901	7
K*	9	932.46376	688.34189	6
G	10	989.48522	561.25475	5
D	11	1104.51217	504.23329	4
G	12	1161.53363	389.20635	3
W*	13	1346.60512	332.18488	2
K	14	1474.70008	147.11340	1

Table S3 – Mass of the tryptic peptide obtained from ME_30

peptide sequence	Mass
MSKELEKVLESSSMA**K**GDG**W**KVMAKGDGWE	
VLESSSMA**K**GDG**W**K	**1494.7257**
ELEKVLESSSMA**K**	**1450.7457**
VMAKGDGWE	**992.4506**
GDG**W**KVMAK	**991.5029**
VLESSSMA**K**	**951.4815**
MSKELEK	**864.4495**
GDGWE	**563.2096**
GDG**W**K	**562.2620**
ELEK	**518.2820**
VMAK	**448.2588**
MSK	**365.1853**

Table S4 – ^{1}H and ^{13}C NMR chemical shifts of the cyclic peptide

Residue	α	β	γ	δ	ε		NH
V	4.37<br>57.8	2.16<br>29.7	0.981;<br>0.965<br>17.20;<br>16.47				
L	4.37<br>52.2	1.56<br>39.1	1.56<br>23.8	0.867;<br>0.908<br>20.57;21<br>.4			8.71
E	4.39<br>52.3	1.97;<br>2.09<br>25.5	2.47<br>29.3	2.11<br>13.4			8.73
S	4.44<br>55.4	3.92<br>60.4					8.45
S	4.45<br>55.4	3.86<br>60.4					8.60
S	4.43<br>55.4	3.83<br>60.6					8.55
M	4.45<br>52.5	2.01;<br>2.11<br>29.7	2.56,2.6<br>1<br>28.9				8.30
A	4.29<br>49.5	1.37<br>15.73					8.37
K	4.75<br>58.02	3.57<br>39.9<br>**CH not CH2**	1.181;1.<br>443<br>23.9	1.747;<br>2.074<br>29.1	2.78;2.8<br>3<br>38.8		8.83
G	3.83	3.54					8.24
D	4.58<br>49.6	2.76,<br>2.92<br>34.9					6.96
G	3.83	3.67					8.06
W	4.78<br>52.8	3.40;<br>3.22<br>25.6	2CH 7.18 (s), 125.1; 3C 107.2; 4CH 7.51 (d), 116.7;<br>5CH 7.24 (t), 119.7; 6CH 7.29 (d) 118.6; 7C 123.0;<br>7aC 135.6; 3aC 127.0.				6.92<br>NH<br>ring:<br>10.42
K	4.41<br>52.4	1.81;<br>1.94<br>29.7	1.46<br>21.8	1.67<br>25.8	2.97<br>38.8		7.60
